# Prediction of RNA Polymerase II recruitment, elongation and stalling from histone modification data

**DOI:** 10.1186/1471-2164-12-544

**Published:** 2011-11-03

**Authors:** Yun Chen, Mette Jørgensen, Raivo Kolde, Xiaobei Zhao, Brian Parker, Eivind Valen, Jiayu Wen, Albin Sandelin

**Affiliations:** 1 The Bioinformatics Centre, Department of Biology & Biotech Research and Innovation Centre, Copenhagen University, Ole Maaloes Vej 5, DK-2200 Denmark; 2 Institute of Computer Science, University of Tartu, Liivi 2-314, 50409 Tartu, Estonia; 3 Quretec, Ülikooli 6a, 51003 Tartu, Estonia

## Abstract

**Background:**

Initiation and elongation of RNA polymerase II (RNAPII) transcription is regulated by both DNA sequence and chromatin signals. Recent breakthroughs make it possible to measure the chromatin state and activity of core promoters genome-wide, but dedicated computational strategies are needed to progress from descriptive annotation of data to quantitative, predictive models.

**Results:**

Here, we describe a computational framework which with high accuracy can predict the locations of core promoters, the amount of recruited RNAPII at the promoter, the amount of elongating RNAPII in the gene body, the mRNA production originating from the promoter and finally also the stalling characteristics of RNAPII by considering both quantitative and spatial features of histone modifications around the transcription start site (TSS).

As the model framework can also pinpoint the signals that are the most influential for prediction, it can be used to infer underlying regulatory biology. For example, we show that the H3K4 di- and tri- methylation signals are strongly predictive for promoter location while the acetylation marks H3K9 and H3K27 are highly important in estimating the promoter usage. All of these four marks are found to be necessary for recruitment of RNAPII but not sufficient for the elongation. We also show that the spatial distributions of histone marks are almost as predictive as the signal strength and that a set of histone marks immediately downstream of the TSS is highly predictive of RNAPII stalling.

**Conclusions:**

In this study we introduce a general framework to accurately predict the level of RNAPII recruitment, elongation, stalling and mRNA expression from chromatin signals. The versatility of the method also makes it ideally suited to investigate other genomic data.

## Background

Regulation of transcription initiation is controlled by several distinct processes, including binding of transcription factors to distal and proximal binding sites and the accessibility of DNA [1-3]. The accessibility of DNA is influenced by chromatin features, including chemical modifications of histones. Modifications of histones and their effect on transcription initiation are the most well-understood chromatin features: acetylation generally is correlated with accessible chromatin, while lysine methylation can have both activating and repressive roles [2]. Histone modifications, as well as other chromatin features, are often referred to as epigenetic marks. We will avoid this term as we are not assessing hereditary changes in chromatin but transient differences in chromatin states between promoters in this study, and instead refer to these as chromatin marks or just histone modifications.

Early studies raised the hope of deriving a "histone code"[4] which based on the occurrence of respective modifications could explain the rate of accessibility, and even predict the locations of different genomic features such as promoters, enhancers, etc. It is only recently that we have had data sets of the size and quality to test whether chromatin marks or DNA signals are in themselves sufficient to predict the location of promoters and enhancers (for example [5-13]), and which marks that are the most predictive. Indeed, several studies have shown that given sufficient histone modification data, it is possible to predict the location of active promoters and enhancers [14-19]. Two recent studies have also shown that the mRNA transcription levels of genes can be predicted by chromatin information around the start site [20,21].

The mRNA level of a gene is essentially a function of its rate of RNAPII elongation and its degradation, but is not necessarily correlated with the rate of recruitment of RNAPII in the core promoter: recent studies [7-9,22] have demonstrated widespread RNAPII pausing near the TSS in mammals and insects. This has regulatory importance since a subset of genes in the studied cell types only have poised, but not elongating RNAPII [8,9,22]. The importance of the poising and/or release of RNAPII make it necessary to distinguish recruitment and elongation of RNAPII from each other and to make separate predictive models for each. Thus, activation of core promoters can indicate the recruitment, release/elongation of RNAPII, or the production of stable mRNA, depending on context.

Therefore, in this study, we extend previous computational efforts by exploring the predictability of RNAPII recruitment, elongation and the release of stalled RNAPII from chromatin signals in the regions around the TSS, taking both the strength of signals and their spatial distribution into account.

We show that RNAPII recruitment, elongation and stalling can be predicted from the chromatin features in the promoter region, and that the positional distribution of marks in the promoter is almost as predictive as the signal intensities of the same marks. Interestingly, when predicting RNAPII stalling/release, binding sites of transcription factors reported to have a key role in this process have less predictive importance than the chromatin signals. We further demonstrate that the significant and joint enrichment of H3K9ac, H3K27ac, H3K4me2 and H3K4me3 is necessary for RNAPII enrichment in the promoter but not sufficient for elongation.

## Results

### A framework for predicting location and usage rate of core promoters

To predict promoter usage from chromatin signals, we constructed a computational framework. We wanted this framework to be able to incorporate any type of signal distributed around the TSS, and take both the signal strength as well as the spatial distribution of the signal into account. Therefore, the -975 to +975 region around human TSSs were divided into 13 sub-regions, each 150 nucleotides (nt) wide, designated "bins", where the center bin was centered on an annotated TSS. While the bin size was originally chosen to mimic the span of DNA wrapped around a nucleosome, our results are robust to changing the number of bins and their position, as described below and in Methods.

**Figure 1:**
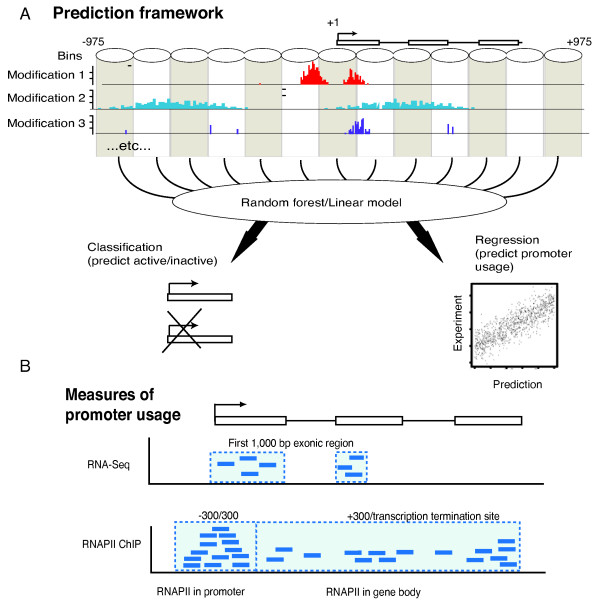
**Measuring and predicting promoter usage**. A) Conceptual framework for prediction of promoter activity. The +/-975 region around annotated promoters was divided into 13 bins where the center bin was centered at the TSS. Within each such bin we count the number of tags per million of available ChIP-seq data, corresponding to enrichment signals of various chromatin marks or other data. These 13 long feature vectors are the input into a Random Forest or linear model to either classify two different sets of promoters from each other or to predict the usage of the promoters measured by various experimental methods (Panel B). B) Illustration of different ways of measuring promoter usage. We measure promoter usage in three different ways: i) mRNA expression by the sum of RNA-Seq tags in the first 1, 000 exonic region downstream to TSS, excluding the tags from the introns; ii) RNAPII recruitment by the sum of RNAPII ChIP tags the region around the TSS (-300~+300) and iii) RNAPII elongation by the sum of RNAPII tags in the gene body (+300~+1, 000).

In each such bin, we counted the contribution from each type of signal by summing the aligned tags from a given ChIP-seq experiment falling into the region; this formed the primary input to the predictive model (Figure 1A). All data used were from the ENCODE dataset and K562 cell line, unless otherwise mentioned (see Methods). For assessing the recruitment and/or stalling of RNAPII we adopted an approach similar to Muse *et al*. [23], counting the number of RNAPII ChIP-seq tags in the promoter region (-300 to +300) since this span will entirely cover the most proximal histones upstream and downstream of the TSS; for measuring elongation we counted the number of tags from ChIPed RNAPII in the downstream gene body region (+300 to transcription termination site (TTS)) (Figure 1B).

We estimated mRNA levels using RNA-Seq tags mapping to the first 1, 000 nt exonic sequence downstream of the TSS, excluding the intronic intervals. This definition was a compromise between the following two factors: i) as RNA-seq reads are randomly sheared and therefore have problems capturing edges of exons and transcripts, we needed a reasonably large RNA space to measure expression and ii) for very long genes, we wanted to avoid counting contributions from possible alternative promoters, or alternative splice forms. The effects of changing the thresholds are described in Methods.

Given this data, we used a Random Forest [24] method for pairwise classification of active vs. silent promoters vs. randomly selected non-promoter regions. Given that a promoter was active we also predicted its usage rate (discussed further below). We defined active promoters (5, 131) as annotated promoters detected by both ENCODE CAGE and RNA-Seq data (see Methods), while we defined silent promoters (2, 838) as the set where neither of the methods detected the promoter.

### Predictive accuracy and feature importance

**Figure 2:**
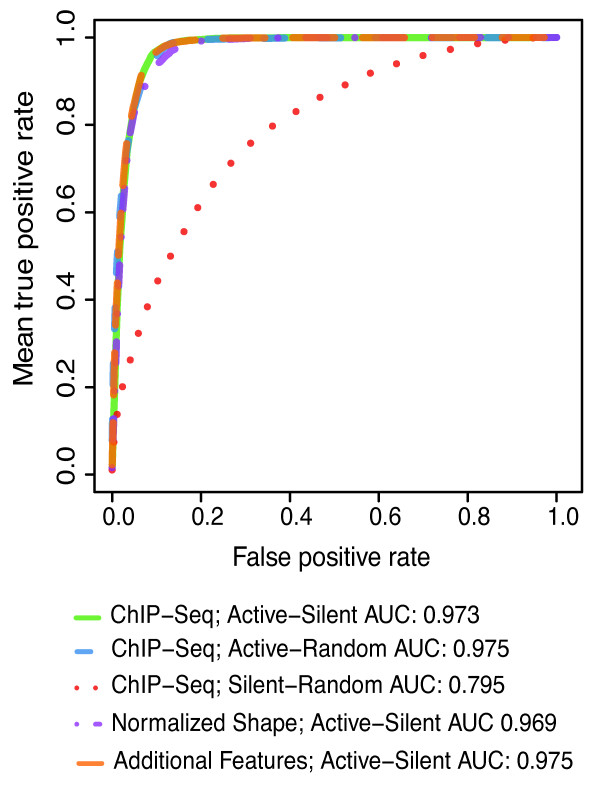
**Promoter activity classification performance**. Receiver-Operator Curves (ROC) were calculated for different 13-bin based classifiers. We made pair wise classifications between active promoters, inactive promoters and randomly selected genomic regions. The performance can be measured by the area under the curve (AUC), where AUC = 0.5 corresponds to a random guessing. Active promoters are easier to distinguish from random genomic background than inactive promoters. Adding additional features (dinucleotide content, DNA methylation status), or only using the positional distribution of marks did not have a substantial impact.

**Figure 3:**
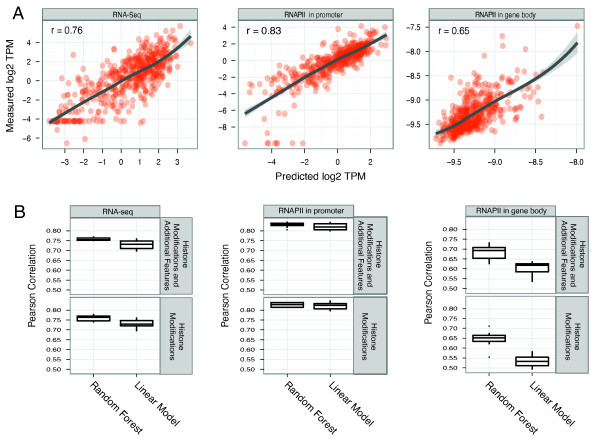
**Predicting the usage of core promoters**. A) Visual representation of the performance of promoter usage rate prediction. We plot predicted *vs *actual promoter usage rates (as measured by RNA-seq in the gene body, RNAPII in the promoter region and RNAPII in the gene body), expressed as log_2 _(Tags Per Million(TPM)). The predicted values are obtained using a linear model with 10-fold cross-validation. B) Summary of promoter usage rate prediction performance. The box plots summarize the correlations by Pearson Correlation Coefficients (PCC) calculated between actual and predicted promoter usage measurements; a perfect correlation will give a PCC of 1. We tested the framework using either only 9 epigenetic modifications, or including additional features (methylation status, dinucleotide and normalized GC content). The variation estimates are achieved performing a 10% holdout experiment on 10 random non-overlapping splits. We used both linear models and Random Forest methods: the Random Forest consistently outperforms the linear model, but the absolute differences in mean PCC values are small (~0.05).

This framework accurately classified active/inactive promoters in terms of mRNA production with an Area Under Curve (AUC)[25] score of 0.973. It attained significantly less precise classifications of inactive promoters vs. random genomic locations (AUC 0.795 and *P *< 10^-16) (Figure 2 and Additional file 1 Figure S1). These accuracies are similar to previous results [14-17,21,26]. The framework is also capable of predicting promoter usage (as opposed to just classifying active/inactive state) in a regression model. Promoter usage can be measured in different ways - recruitment of RNAPII, elongating RNAPII, or by the concentration mature mRNAs. For recruitment and elongation of RNAPII, we predicted the density of RNAPII ChIP tags in the regions around the TSS and in the gene body, respectively, and achieved mean Pearson Correlation Coefficients (PCC) of ~0.83 for RNAPII around the TSS and ~0.65 for RNAPII in the gene body by only considering the histone modification patterns. Similarly, we could predict mRNA expression based on RNA-Seq tags with a mean PCC of ~0.76 (Figure 3A, B).

The mRNA regression results are similar to those of previous studies [20,21], which used microarrays or RNA-Seq, but an important difference is that we, in contrast to previous studies, removed transcriptionally silent genes before the analysis. This is important since many genes are transcriptionally silent, and therefore the training sets will be unbalanced unless these are removed.

We wanted to see if our results were dependent on our specific methods and thresholds. First, we applied linear regression models (see Methods) to compare the results with the Random Forest method. Although the correlation scores obtained using Random Forest were consistently (and statistically significantly, *P *< 0.05, see Methods and Additional file 1, Table S1) better than the ones obtained with standard linear regression, in absolute terms the mean PCC difference was small (~0.05)(Figure 3B and Additional file 1 Figure S2). This indicates that the framework is robust and that different types of machine learning models can be successfully applied to it. We also explored the effects of changing the definitions of promoter and gene body regions for both RNAPII and RNA-seq data (See Methods and Additional file 1, Figure S3 and Additional file 1, Table S6-7), and found that while the definitions can influence the PCC values, the absolute differences are not large, ~0.01-0.02 for RNPII measurements and ~0.04-0.1 for RNA-Seq.

**Figure 4:**
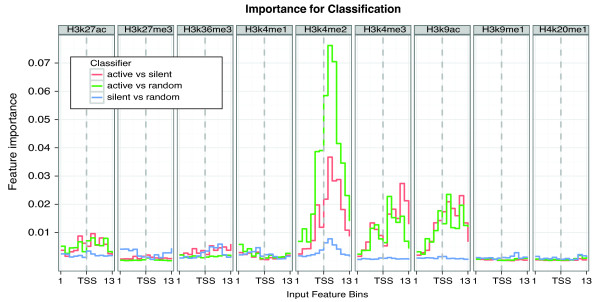
**Feature importance for promoter classification**. We show the importance of each histone mark and positional bin for the classification between active promoters, silent promoters and randomly selected non-promoter regions. The importance is computed as the mean decrease in accuracy as defined by the Random Forest method, where high values indicate high importance for a particular feature in the prediction. The X-axis denotes the entire set of 117 input features, consisting of 13 bins per epigenetic mark where the middle bin corresponds to the 150 nt region around the TSS (See Figure 1A). The results show the high influence by H3K4me2, H3K4me3 and H3K9ac in distinguishing the active promoters.

An advantage of the Random Forest method in comparison with Artificial Neural Networks [27] or Support Vector Machines [28] is that the importance of each input feature for the final prediction can be easily assessed, which can give insights into the mechanisms underlying the input data. As expected, H3K4me2, H3K4me3 and H3K9ac signals have the largest importance on classification of inactive vs. active promoters, especially H3K4me2 (Figure 4). The most informative signals are located immediately around the TSS. This is consistent with previous studies establishing that H3K4 di- and tri-methylation are indicative of active promoters [10,29].

**Figure 5:**
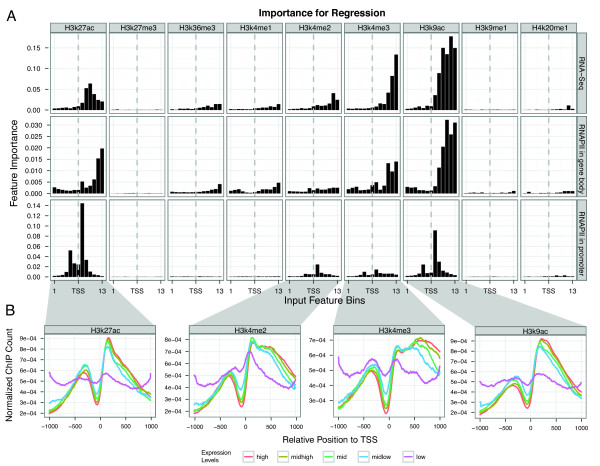
**Feature importance for prediction of promoter usage level**. A) Importance of features. Similarly to Figure 2A, we assessed the importance of the 117 features for the prediction of promoter usage level, based on three different promoter usage measurements: RNA-Seq and poised and running RNAPII. The importance (Y-axis) was measured by the influence of the feature on the mean square error. The prediction of RNA-Seq and RNAPII of the gene body density show similar patterns of importance where the downstream ChIP-Seq signals of the activating marks appears to be more informative. On the other hand, the acetylation marks seem to be most important in predicting the RNAPII recruitment, especially the first bin after the TSS, corresponding to the +1 nucleosome. B) Positional distributions of the four most informative histone marks broken up by expression. The promoter set was divided into 5 classes depending on mRNA expression using RNA-Seq data. Within each class, we normalized the counts for H3K4me2, H3K4me3, H3K9ac and H3K27ac in all bins to only retain the shape of the distributions. For the methylation marks, there is more variation in the distributions downstream of the TSS than upstream. The two acetylation marks have the highest relative signal around the position for the +1 nucleosome, while the methylations have high signals for ~5 downstream nucleosomes.

When predicting the promoter usage level we observe roughly the same marks being important as in the active versus inactive classification (Figure 5A), with a few interesting differences. Firstly, the importance of H3K27ac and H3K9ac for the prediction of promoter usage level is substantially increased compared with H3K4me2 and H3K4me3. This fits well with the hypothesis that the acetylation marks are indicative of the scaling of promoter usage while the H3K4me2 and 3 marks function more as platforms to establish the promoter [30].

Secondly, when predicting RNAPII density within the gene body or mRNA production by RNA-Seq, the chromatin signals are generally more informative downstream of the TSS. In contrast, signals located upstream and downstream of the TSS are important for the prediction of the RNAPII density in the promoter.

To investigate this further, for each promoter, we normalized the contribution of each input feature *S_i, j_*, where *i *is the ChIP experiment (such as H3K4me3)and *j *indicates the bin:

(1) $$\text{Norm(}{\text{S}}_{\text{i,j}}\text{)}=\frac{{\text{S}}_{\text{i,j}}}{\underset{\text{j’ = 1}..\text{13}}{\sum }{\text{S}}_{\text{i,j’}}}$$

This normalization will retain the shape of the distribution of each modification in a single promoter but not the overall magnitude of signals. We then plotted the normalized distribution of H3K4me3, H3K4me2, H3K9ac and H3K27ac for the promoters broken up by their level of mRNA expression (Figure 5B). From these plots, we can see there is little variance between the different expression classes in very immediate regions around the TSS but a high variance between H3K4me2 and me3 in the +500 to +1, 000 nt region, corresponding to the third to sixth nucleosome downstream of the TSS. However, this is not true for the two acetylation marks (Figure 5B). These observations indicate that not only the signal strength of the marks but also some parts of their positional distributions are informative. This observation encouraged us to investigate how much predictive power there is in the positional distributions as described below.

### The distribution of histone modifications is both predictive for defining the promoter and for determining expression level

Several studies have shown that several histone marks have characteristic patterns around promoters - for instance, H3K4me2 and me3 marks tend to have a double peak around active TSSs and flanking the nucleosome-depleted region at active TSSs [6,31,32]. Since the model we have takes both the signal position and strength (sum of tags) into account, we wanted to see if the positional distribution in itself had predictive power disregarding the signal strength. Therefore, we normalized the contribution of each feature within each promoter as described in Equation 1.

Using the normalized data, we tried to distinguish active and inactive promoters from each other and predict the usage rate of the active ones. Classification using only the distribution shape gives an AUC value of 0.969, compared to 0.973 (Figure 2) when also using the signal strength information, showing that the shape of the distribution alone is highly indicative of promoter activation.

Regression using only the positional distribution gave a PCC for RNA-Seq of 0.67 compared to 0.76 when also using the signal strength. For RNAPII in the promoter, the corresponding values are 0.77 and 0.83 and for RNAPII in the gene body RNAPII 0.41 and 0.65.

While the regression results using signal strength were always significantly better than using only the normalized shape as input (*P *< 0.05 in all cases, see Methods and Additional file 1, Table S2), it is clear that the distribution shape of histone marks has substantial predictive power.

Increasing the number of bins in general only gave minor improvements (Additional file 1, Table S3). A caveat with this analysis is that the shape and the signal strengths are not strictly independent, as more complex distributions require higher number of ChIP tags mapping into the region.

### Incorporating additional features

We tried to improve the regression performance by also incorporating more elaborate models and additional data. The Random Forest method takes pairwise interactions between features into account, while the linear method does not. To see if this could explain the lower accuracy of the linear method we included all possible pairwise interactions in the linear model. This did not improve the performance of the linear regression model substantially (Additional file 1, Figure S4), indicating that an assumption of independence of the marks is a reasonable simplification for modeling promoter usage by histone marks.

Encouraged by the fact that high and low CpG content promoters are subject to different histone modifications and methylation patterns, we included extra features such as dinucleotide content, GC content, normalized CG dinucleotide fraction [33] and DNA methylation status to the analysis, but these features only improved the predictability slightly (Figure 3B, Additional file 1, Table S3), and the difference compared to the original analysis is not statistically significant(Additional file 1, Table S4). This suggests that the chromatin signals indirectly incorporate this information, although the causality is unclear.

### Exploring the necessity of histone marks for recruiting RNAPII

While the feature importance from the Random Forest can give important hints on the biological properties of the system, it cannot be directly translated to ascertain whether a particular mark is necessary since the redundancy of marks is not considered. Since we identified H3K9ac, H3K27ac, H3K4me3, and H3K4me2 to be the most important features for predicting the amount of RNAPII at the promoter, we proceeded to analyze if there is a strict requirement to have these signals present or if some of the marks are optional but their presence contribute to the overall RNAPII recruitment or elongation.

To do this, we needed a threshold defining whether a certain mark is enriched or not. We used a threshold based on the 95^th ^quantile of the signal strength distribution from randomly chosen genomic regions for respective marks, and also for the enrichment of RNAPII respectively in the promoter and in the gene body (see Methods).

**Figure 6:**
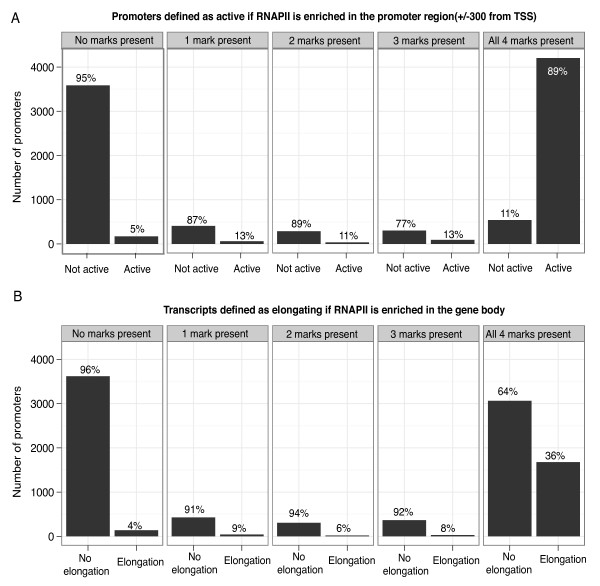
**Necessity of H3K4me2, H3K4me3, H3K9ac and H3K27ac in RNAPII recruitment**. A) We defined thresholds based on 95 percentiles for the presence of H3K4me2, H3K4me3, H3K9ac and H3K27ac marks in the promoter region(+/-1000 nt), and then counted how many promoters that have 0, 1, 2, 3 or all marks. For each group we counted how many of the promoters that had RNAPII signals exceeding the threshold in the vicinity of the TSS(+/-300). In each subplot the left bar represent the promoters that have no RNAPII present while the right bar represents those that have RNAPII present. B) We repeated the same analysis in panel A, but instead counted how many genes that had higher RNAPII signal in the gene body than expected by random. We note that most promoters either have no marks or all four marks. Furthermore, ~95% of the promoters with no marks present have no RNAPII signal, while 89% of promoters with all marks have significant signals for RNAPII in the promoter. This tendency is not as strong when measuring RNAPII in the gene body, suggesting that the four marks are necessary for recruitment but not sufficient for the elongation of RNAPII.

We next investigated the impact of co-occurrence of H3K9ac, H3K27ac, H3K4me3, and H3K4me2 for RNAPII occupancy, measured by RNAPII density in either the core promoter or in the gene body. We subdivided the set of 9, 693 non-overlapping transcripts (see Methods) depending on whether they have none, some, or all of the above marks present. This analysis showed that most promoters either have none or all the marks enriched, which fits with the finding that H3K4me3 and AcH3 marks are prone to be more stable when they occur together [34]. Moreover, 90% of the promoter regions are lacking RNAPII signal unless all marks are present; conversely, if all marks are present, 89% of the promoters regions have significantly enriched RNAPII signal (Figure 6A). This pattern is not present when assessing promoter usage by the density of RNAPII in the gene body, where only 36% of the core promoters with all marks contain RNAPII in the gene body (Figure 6B). This is consistent with some promoters only having recruited, but not elongating, RNAPII (further discussed below). Varying the thresholds to the 90^th ^or 99^th ^quantile, or using a Poisson-based test with *P *< 0.05 or *P *< 10^-5 ^significance thresholds as used in Ernst *et al*.[19] did not change this trend (Additional file 1, Figure S5A).

Since the K562 cell line is known to have specific chromosomal aberrations [35,36], we wanted to ensure that these properties observed were general. Therefore, we redid the analysis for the set of all promoter regions using ChIP data from two other cell lines, NHEK and HUVEC from the ENCODE set, which gave consistent results (Additional file 1, Figure S5B).

This implies that all of the four marks are necessary in the recruitment of RNAPII at the TSS but not sufficient for elongation. A necessary caveat is that this causality (histone marks causing recruitment) cannot be proved rigorously since i) these results could be explained by a third confounding variable that always co-occurs with the four marks, ii) we are not measuring a single cell and we do not know if the marks physically co-occur on the same nucleosome and iii) we do not know if the histone modifications are needed to recruit RNAPII or vice versa.

We hypothesized that some of these modifications occur simultaneously. This fits with a recent study where Pasini *et al*. [37] showed that recruitment of EZH2 to the promoter leads to tri-methylation of H3K27 and prevents H3 acetylation in polycomb group target genes, especially H3K27ac but also H3K9ac, forming a methylation-acetylation switch. This model would predict that H3K27me3 would be negatively correlated with H3K27ac and H3K9ac; our data supports this (Spearman Correlation Coefficients (SCC) < -0.2); in fact, since the four marks under consideration are highly correlated (SCC > 0.8), all are negatively correlated with H3K27me3 (Additional file 1, Figure S6).

### Predicting stalling and release of RNA polymerase II

Several recent studies have shown that a substantial set of promoters recruits RNAPII, which is not released for elongation [38,39]. This is important since it indicates that the recruitment of RNAPII might not always be the rate-limiting step of mRNA transcription. Thus, it is worthwhile to investigate the associations between the modification status of histones and the RNAPII stalling characteristics.

A possible solution is to correlate the elongation rate of RNAPII with the amount of downstream marks that are found in the transcribed regions, such as di- and tri-methylation of H3K36 and H3K79me2 shown by earlier studies [5,10,40-42]. However, these marks are likely deposited as a consequence of elongation, and it is less likely that the initiation complex is directly influenced by the histone marks in the gene body. Therefore, we wanted to investigate if the stalling features of RNAPII could be predicted by chromatin signals around the TSS.

The amount of stalling vs. elongation has typically been measured by taking the ratio between the density of RNAPII at the core promoters vs. in the gene body - called either the travelling ratio or the stalling index(S index)[22,26]. Here, we used the S index as defined in Muse *et al*. [23]:

(2) $$S=log_{2}\left(d\left(RNAPII_{promoter}\right)\right)-log_{2}\left(d\left(RNAPII_{body}\right)\right)$$

where *d *is the number of RNAPII ChIPed tags per nt in the given region. This will give a value between ~-2 and ~4 (Additional file 1, Figure S7). We defined the promoter region as +-300 region around the TSS since the span will entirely cover the most proximal histones upstream and downstream, and defined the gene body to be the remaining part of the gene.

We then tried to predict the S index of the 9, 115 genes (see Methods) by using the 13 bin framework as above, and compared predicted and actual S index values. We achieved a mean PCC of 0.83, similar to our previous regression results. Addition of dinucleotide densities as additional features only resulted in a slight improvement (PCC = 0.85).

We reasoned that while the epigenetics data clearly has substantial predictive power by itself, including additional features might increase this value even further. Several transcription factors are known to be correlated with polymerase elongation, including the negative elongation factor NELF that pauses the elongation of RNAPII [43], and the transcription factor cMyc [11], that has a role in regulating the release of paused RNAPII. Thus, we also tried to include ChIP data for NELFe, an important subunit of NELF for the inhibitory function, and cMyc in our models to improve the prediction power.

**Figure 7:**
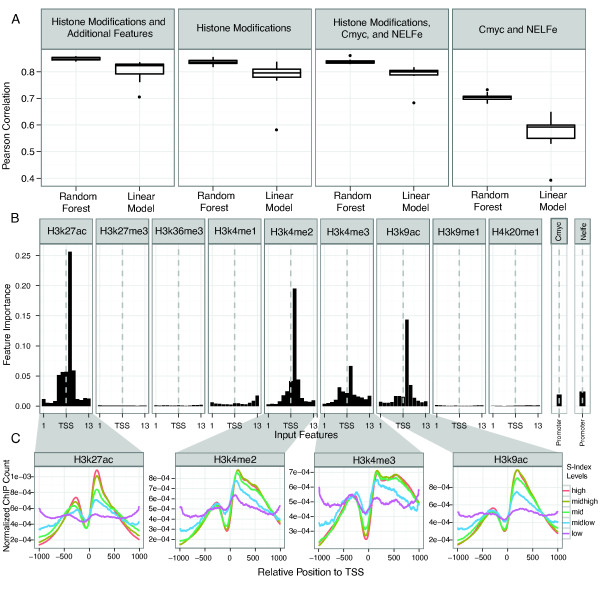
**Distinguishing of RNAPII stalling/elongation state using chromatin signals**. A) Correlations between the observed and predicted S index. The box plots summarize the variation estimated by 10 times cross-validation for both Random Forests and linear models. The regression was done on four different feature sets: 1) All 9 histone modifications as well as methylation status, dinucleotide content and normalized GC content 2) All 9 histone modifications 3) All 9 histone modifications the transcription factors cMyc and NELFe 4) only cMyc and NELFe. B) Feature importance in S index prediction. The importance of most marks for regression of stalling index is increasing in the first bin after the TSS: this is also seen in the regression of poised RNAPII in the promoter (Figure 2B). NELFe and cMYC (assessed as an overall signal within the promoter region) have substantial, yet lower predictive power compared to the chromatin data. C) Positional distributions of the four most informative histone marks broken up by S index. The promoter set was divided into 5 classes depending on S values. Within each class, we normalized the counts for H3K4me2, H3K4me3, H3K9ac and H3K27ac in all bins to only retain the shape of the distributions. The region that displays the most variance between the classes corresponds to the position of the first nucleosome downstream of the TSS.

Surprisingly, the prediction based on only cMYC and NELFe alone gave a lower PCC of 0.7. The lower amount of information in the transcription factor binding sites *vs *chromatin signals could be due to several reasons. Firstly, a recent study has shown that release-associated factors such as cMyc can be found in both stalled and elongating promoters [44]. Secondly, their interaction with the pre-initiation complex might not require strong binding to DNA; if so, the factors would not be detected as clear ChIP peaks in the proximal promoter. Notably, using these two factors as additional features together with the chromatin data only improved the regression results slightly (Figure 7A and Additional file 1, Table S5). We hypothesize that the predictive power of these factors are to a large degree already contained in the histone mark data. Similarly, sequence patterns such as dinucleotide content, including CpG, or the presence of TATA-boxes had limited predictive power (Additional file 1, Figure S8).

The most informative epigenetic features for the regression are still H3K27ac, H3K9ac, H3K4me2, and H3K4me3 with a clear preference for the first bin after the TSS (Figure 7B), suggesting that modifications within the +1 nucleosome are directly or indirectly associated with the stalling/release decision. Indeed, if we plot the normalized mean ChIP density of different promoters divided by the S Index (Figure 7C), we observe that the highest difference between the classes is just downstream of the TSS, and not further downstream as observed when predicting the elongation rate (Figure 5B).

Since the same marks are indicated to be important in the stalling prediction as in the elongation prediction, we wanted to see whether we predict the S index as a by-effect of predicting the elongation. Therefore, we split the testing promoters by expression level into five classes by RNA-Seq and predicted the S index for each subset by using the original model (Additional file 1, Figure S9). This gave PCC range between 0.65-0.73. The low variance between the results from the expression subsets suggests that the results are not simply an effect of predicting the mRNA level, consistent with the differences in feature importance between the two predictions discussed above.

To ensure our framework is general and not over-fitted to the K562 cell line, we applied the same model (trained on K562 data) to predict the *S *index of RNAPII data in the HUVEC cell line within the ENCODE set. By using the corresponding ChIP data as the model input, we reached a PCC of 0.71 (Additional file 1, Figure S10) which is slightly lower than that of the originating cell line. This is likely due to normalization issues since the total counts of chromosomal signal enrichment as well as the sequencing depths are different between the experiments.

## Discussion

A few recent studies have demonstrated that epigenetic data is predictive of the production of mRNAs as measured by microarray and RNA-Seq [20,21]. In this study, we have expanded this into predicting RNAPII recruitment, elongation and stalling, as well as assessing the impact of the distribution of signals *vs *the signal strength.

Predictive methods are even more usable if they are not "black boxes" and can identify what biological features are the most important for accurate predictions. Previous computational methods identified different sets of histone marks to be the most predictive of mRNA production; Karlic *et al*. found H3K4me3 and H3K79me1 to be most informative in predicting the expression level in low CpG content promoters whereas the expression in high CpG ones depend more on H3K27ac and H4K20me1 [20]. In another study by Cheng *et al*., H3K4me2 and H3K79 sets are reported to be more predictive than RNAPII in predicting the gene expression [21]. This difference could in part be due to the set of marks used as input in both studies not being identical, but could also be due to a redundancy in the chromatin signals around promoters. While the redundancy makes predictions easier, it makes the interpretation of the predictive features harder in terms of causality.

In our study, we have broken up RNAPII recruitment and elongation and found that the most predictive marks for both the processes are H3K4me2, H3K4me3, H3K9ac and H3K27ac, but the location of the marks have different predictive importance for respective processes. In fact, the positional distributions of marks are almost as informative in the prediction as the signal strength (the number of ChIP tags). In agreement with a previous experimental study [17], the methylation marks are more predictive of identifying promoters (the location of active promoters) while the acetylation marks become more important when predicting the amount of RNAPII recruitment and elongation. There are also differences in spatial information in these two processes: the downstream variances of both methylation and acetylation marks are more important than those upstream and around TSS for the RNAPII elongation and mRNA prediction, while the opposite is true for predicting RNAPII recruitment.

However, a follow-up analysis showed that almost all promoters with high amounts of recruited RNAPII have all these marks simultaneously enriched. This fits well with previous studies based on individual correlations - for instance, Kim *et al *found that 90% of all promoters having a RNA polymerase II pre-initiation complex (PIC) also contain acetylation of H3 and/or H3K4me2 [45]. Consistently, Wang *et al *found a modification backbone of 17 modifications that co-localize in ~25% of human promoters and only a very few promoters have a subset of these modifications [17]. All our 4 modifications are part of this backbone. However, it is important to point out that the prevalence of a signal and its importance in prediction of RNAPII recruitment or elongation is not necessarily the same: if a signal occurs at all promoters regardless of its level of RNA recruitment, it will have little predictive importance.

While our study showed that all of the four marks have to be present for RNAPII recruitment in three different cell lines, the causality or order of recruitment is not clear. The study by Wang *et al *showed that H3K4 methylation is needed for acetylation of H3K9 and that both marks are required for the recruitment of RNAPII, but not sufficient for the elongation [46]. However, one could also envision a process where the RNAPII or the PIC will recruit enzymes responsible for the modification of the histones, as suggested in [47,48].

In addition, among these four marks, H3K4me2/3 have been reported to be respectively present in 97% and ~ 75% of all promoters in human cells, but only ~ 50% of these promoters produce detectable transcripts [9], indicating that they by themselves are not predictive of elongation of RNAPII. In this study, we have shown that the ratio between stalled and elongating RNAPII can be predicted from chromatin signals around the TSS (ignoring signals in the gene body). Consistent with the above, the acetylation signals, in particular H3K27ac, are the most informative for predicting the stalling index.

It is surprising that adding ChIP data for the transcription factors cMyc and NELF, known to be involved in the release of stalled RNAPII, does not give a substantial improvement in the prediction; in fact, if only using the ChIP data from the transcription factors in the proximal region, the prediction results are much lower, suggesting the detected binding sites of these two factors are not very informative. One possible reason for this is that the histone marks or other chromatin signals capture the effect of these factors. Alternatively, the interaction between these transcription factors and RNAPII might not be detected by the ChIP experiments.

## Conclusion

The field of genomics is now in a situation where large data sets can be produced with small effort and cost compared to previously, meaning that the challenge has shifted towards analyzing and understanding the data produced. For this, we need frameworks that are both flexible, easily used and that can systematically mine the data to produce viable hypotheses to understand the underlying biology. In this study we have shown the feasibility of predicting RNAPII stalling, transcription and mRNA production at the core promoter level using a relatively simple machine learning framework which can also suggest new biological mechanisms, or reinforce previous hypotheses in a more statistically rigorous way.

We have found that the spatial distribution of marks is almost as predictive as their signal, and that different parts of the promoter are informative for prediction of the recruitment, elongating and release of RNAPII. Moreover, we find that the four marks, H3K4 di- and tri- methylation, H3K9ac and H3K27ac are nearly always co-occurring in promoters where RNAPII is recruited. Among these marks, H3K4 di- and tri- methylation are more informative for determining the promoter position whereas acetylation marks are more predictive of the amount of promoter usage.

## Methods

### Data and post processing

All primary data was downloaded from the ENCODE UCSC browser [49,50]http://genome.ucsc.edu/ENCODE/. Only data labeled as unrestricted (9 months after release date) were used.

### Cell lines

We made the main analysis using the K562 cell line data and used HUVEC and NHEK data for validation, all from the NCBI36 (HG18) assembly.

### Gene models

We used the UCSC known gene track [49,50] as gene models and for promoter annotation, unless specifically described below.

### Core Promoter set

All TSSs were derived from the gene track mentioned above. Since the alternative transcripts may cause ambiguous cases when measuring the tag expression, we only used transcripts that do not overlap any other transcript from the track. This gave a set of 12, 872 core promoters. Due to that we measure the mRNA expression by RNA-Seq in the downstream 1, 000 exonic regions (excluding the signals from intronic intervals), all the transcripts that were not long enough were discarded, resulting in a 9, 115 set for final analysis.

### ChIP-Seq data of histone modifications and RNAPII

RNAPII as well as the histone modification data for H3K4me1, H3K4me2, H3K4me3, H3K9ac, H3K9me1, H3K27ac, H3K27me3, H3K36me3, H4k20me1 in all the three cell lines were downloaded from 'Broad Histone' tracks [34,37,51] in the UCSC browser as mapped reads. We applied the MACS peak finder [52] on these datasets with standard settings but vfold = 10, using the input control ChIP for respective cell as background and used the produced 1 nt resolution wig files of the shifted tags (not the peaks) as our basal data. Replicates were pooled. These tracks are produced by the Broad and Bernstain laboratories and released for public use.

### Cap Analysis for Gene Expression (CAGE) data and processing

We used the nuclear CAGE libraries from the K562 cell lines from the 'ENCODE RIKEN RNA Subcellular Localization by CAGE Tags' track in the UCSC browser. These tracks are produced by the RIKEN Omics Science Center [53-55]. The data was transformed so that we count the sum of 5' ends of reads at each genomic nucleotide, given strand, as in [56]. The tag counts from the immediate vicinity (-75/+75) around the TSS, normalized to tags per million were calculated for measuring the promoter usage.

### RNA-Seq data and processing

We used K562 Tier 1 polyA+ RNA-Seq data produced by the Snyder laboratory [57-59] from the 'Yale RNA-Seq' track in the UCSC browser. Only the tag counts from the first 1, 000 exonic nt downstream of each core promoter were summed and normalized to TPM scale, which we used as an estimate of the amount of produced RNA from that promoter. Genes with a total exon length shorter than 1, 000 nt were excluded from further analysis. i). We tested the effect of varying this definition to the first 500 nt or all exonic nucleotides (Additional file 1, Table S6); this resulted in PCC values between 0.7-0.75 and 0.6-0.67, respectively, which are both significantly (*P *< 0.05) lower than when using the definition above which typically gave PCC scores of 0.75 or higher. While the decrease is not large in terms of absolute numbers (~0.4-0.1 difference in mean PCCs), it probably reflects the issue discussed above - the shorter definition might reflect the issues with detecting exon edges while including all known exons increases the risk of not capturing relevant splice form or erroneously the contributions of unannotated downstream alternative promoters.

### Data for transcription factors

We downloaded the ChIP-seq c-Myc and NELFe data from the K562 cell line from the ENCODE 'Open Chromatin' [60,61] track in the UCSC browser. The total signal in the +/- 1000 nt region around the TSS, normalized to tags per million was used as a single feature for the predictor.

### Methylation data

We downloaded the ENCODE methylation data for K562 from the Hudson Alpha lab [62] from the UCSC genome browser. The data contains the methylation status for all CpG regions in the genome. The methylation status for each bin was set to "methylated" if just one basepair in the bin was methylated and not methylated otherwise. In this way the methylation status was used as a binary feature in the predictions.

### Dinucleotide content and normalized GC-content

We extracted the promoter sequences for all used genes and divided them into bins. For each bin we counted the number of occurrences of each dinucleotide and divided by the length of the bin-1. These 16 numbers for each bin were used as input features in the prediction. The normalized GC-content was computed as defined by Saxonov *et al*. [51].

### Overall framework for capturing genomic signals around TSSs

To retain the positional distribution as well as signal strength as inputs we separated the +/-975 nt regions around the TSS into 13 150 nt wide bins. Starting from setting up the center bin +/- 75 around the TSS, flanking ones were gradually extended towards upstream and downstream. Given a bin and a ChIP dataset, we counted the number of TPMs from the ChIP data set mapping to the region. This results in the size of an initial feature set (number of bins)*(number of data sets).

These thresholds were selected based on biological and practical reasons. The +-975 region was mean to encompass the core promoters as well as its flanking regions. The reason for not using +-1000 is that the region has to be dividable by the 150 nt bins, whose sizes was chosen not only for the practical reason that it gives a reasonable number of bins, but also as it roughly corresponding to the area occupied by a nucleosome. However, as described in the main text, changing the number of bins and thereby their positions has no substantial impact on the results. Likewise, as shown in Figure 2, most of the predictive signals reside in the bins around the TSS, so changes in the overall region investigated will not have substantial impact as long as this region is included. It should be noted that two similarly scoped papers discussed in the main text [20,21] used much larger regions (+-2000 nt around TSS) and did not report higher correlations when predicting expression.

### General description of prediction models

Given the above input feature framework, we constructed two types of predictive models: one for classification (typically between two types of promoters) and one for regression (typically for predicting promoter usage given chromatin signals).

All the classifications were made by using the Random Forest method [24], as implemented in the RandomForest R package [63]. ROC curves were drawn using ROCR [64]. The feature importance in the classification problems was calculated as the mean decrease in accuracy.

For performing regression we used Random Forest and four different versions of linear regression. The linear models included ordinary linear regression as implemented in R function lm and regularized versions of it, namely: ridge-, lasso- and elastic net regression. These three methods are designed to prevent over fitting and perform feature selection when the number of predictive variables is large. We fitted these linear models using glmnet [33] package in R with parameter alpha valued at 0, 1 and 0.5 to achieve correspondingly the ridge-, lasso- and elastic net regression. The regularized models produce a sequence of model fits corresponding to different values of the regularization parameter lambda. In this case we chose the model showing the best correlation with the training data. All the other parameters were kept as default in the analysis. The regression using Random Forest was performed with RandomForest [63] package in R using the default settings. We used the mean decrease in mean standard error (MSE) to assess the importance of features in Random Forest model. The resulting importance from the multi-folds cross-validation was calculated as the average of the individual values.

### Classification of promoter activity

We classified active, inactive and randomly selected non-promoter regions from each other using chromatin signals as inputs, as described below.

### Definitions of promoter sets for classifications

The active promoter set (5, 131 promoters) was defined as +/-1, 000 nt regions containing both CAGE and RNA-Seq tags. We considered only genes that were long enough (exonic length of 1000nt or more) for a reliable RNA-Seq density measurement.

The inactive (or silent) promoter set (2, 838 promoters) was defined as promoters with no tags from either CAGE within +/-75 nt around the TSS or RNA-Seq in the first 1000 nt exonic region. We selected random genomic regions of the same size for the random position set.

### Training and evaluations for classifications

For training and evaluating the results for the classification, we used a hold-out strategy wrapped by 10-fold cross-validation. In order to minimize the bias from unbalanced sizes of the binary classes, we randomly selected the same amount of data from the larger class according to the size of smaller class in each run of the cross-validation. Then with two equal-sized classes, we further divided the data for training and testing by the proportion of 70% and 30%. The local AUC and importance for one fold was evaluated from the performance of the trained model in the test set. After finishing 10-fold repeats, the overall AUC and importance were calculated as the mean of the results.

### Expression measurements used as responses in regression

For predicting the expression levels we considered only the active promoters used in the classification, described above. We applied log2 transformation to the data in order to make it more suitable for the regression task. To avoid taking the logarithm of 0, we added a pseudo count of 0.001 to both input features and output.

### Training and validation for regression

We assessed the performance of the predictions using a repeated hold-out scheme. At each step we randomly divided the dataset of 5, 131 promoters defined above into 10 equally sized parts. Then we trained the model using the 9 proportions of them and tested the model predictions on the exclusive part. We train on the data 10 times until all of the subset had been used as a test set. For evaluations, we calculated the Pearson Correlation Coefficients between predicted and actual log_2 _TPM values. This way we could estimate both the regression accuracy and also how stable the accuracy is, which is important when comparing the results from different methods.

### Assessing interactions between input features

To study the influence of interactions on the expression level prediction, we used linear models with interaction terms. In our original dataset we divided the promoter into 13 bins, to assess the positional influences of the modifications. However, including interactions between all these parameters would make the model too large. Since we achieve almost as good result using 1 bin per modification as 13 in the regression problem, we used only 1 bin per mark for testing interactions. We then tested the model using a cross-validation schema as described above.

### Regression of the stalling index

The stalling index value *S *were calculated as described in the main text. For the regression of s index, we randomly selected 30% of the data from the 9, 115 set as the testing data and used the rest in the training procedure. We then used the same 13-bin prediction framework and methods as we used in the previous regression problems. In addition, c-MYC and NELFe ChIP-seq signals were also used as optional input features.

### Thresholds for histone marks

To be able to say with confidence if a promoter has a specific histone mark present we need to assess the random expectation of tags from the given mark in a genomic region of the same size. We sampled 33.000 random genomic regions and counted the number of tags for each mark in each region. We set the threshold to the 95^th ^quantile of the random distribution meaning that 5% of the random regions would be considered to have the mark present. Thresholds for RNAPII present in the promoter region and gene body were defined in the same way. As mentioned in the text, we also varied the threshold to the 90^th ^or 99^th ^percentile, and also tested the Poisson based methods as the one also used by Ernst *et al*. [19], with a significance threshold of *P *< 0.05 or *P *< 10^-5^.

### Assessing the significance of the different performance between prediction models

For measuring the significance of the difference between different classification tasks, a list of p values for one particular pairwise classification was computed in each fold of the cross-validation procedures, by the Hanley and McNeil test [46] implemented in the R package MKmist [40]. Both the original AUCs and the AUCs recomputed in test were based on the same posterior probabilities estimated from the corresponding Random Forest models. For evaluating the difference between different regression tasks, we applied two-sided t test on the resulting PCCs obtained from cross-validation.

### Determining the presence of TATA box for each promoter

We predicted TATA-boxes in the -50 to -10 region of each TSS using the TATA-box positon weight matrix from the JASPAR database [65] and a score threshold of 70% (as described in [38]). If one or more sites were predicted, the promoter was labeled as TATA-box containing.

### Visualization

We made all plots in R using the ggplot [66] and ROCR packages [64].

## Competing interests

The authors declare that they have no competing interests.

## Authors' contributions

YC, MJ, RK, EV, XZ and AS performed the analysis and interpreted results. BP and JW assisted with statistical issues. YC, MJ, RK and AS made all figures. All authors wrote the manuscript. All authors read and approved the final manuscript.

## Supplementary Material

Additional file 1 **Supplementary figures with legends**. This file contains Supplementary Figure S1-S10 and Supplementary Table S1-S6 Click here for file
