# Supplementary material for: Prediction of RNA Polymerase II recruitment, elongation and stalling from histone modification data
Source: BMC Genomics. 2011 Nov 3;12:544. doi: 10.1186/1471-2164-12-544 (PMC3228824; doi:10.1186/1471-2164-12-544)
Supplement: Additional file 1 — Supplementary figures with legends. This file contains Supplementary Figure S1-S10 and Supplementary Table S1-S6 [file 1471-2164-12-544-S1.PDF]

SUPPLEMENTARY MATERIAL TO

Prediction of RNA Polymerase II recruitment, elongation and stalling from histone modification data

Yun Chen[1], Mette Jørgensen[1#], Raivo Kolde[2,3#], Xiaobei Zhao[1], Brian Parker[1], Eivind Valen[1], Jiayu Wen[1], Albin Sandelin[1*]

[#] Shared second authors; * Corresponding author

[1] The Bioinformatics Centre, Department of Biology & Biotech Research and Innovation Centre, Copenhagen University, Ole Maaloes Vej 5, DK-2200 Denmark

[2] Institute of Computer Science, University of Tartu, Liivi 2- 314, 50409 Tartu, Estonia.

[3] Quretec, Ülikooli 6a, 51003 Tartu, Estonia.

This material contains: 10 Supplementary Figures and 7 Supplementary Tables

Supplementary Figure 1

Significance of the AUC difference in Figure 1C

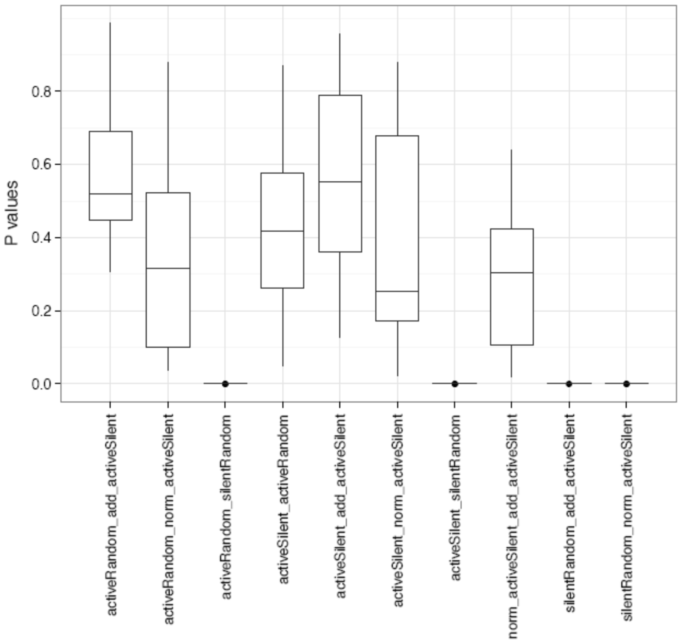

Supplementary Figure 1

The plot shows a boxplot of the distributions of the significance scores when comparing the differences between the AUCs in **Figure 2**. The Y axis shows the *P*-values calculated by the Hanley McNeil's test [1] from each fold of the cross-validation procedure (see Methods for details). It is evident that all AUC pairs are not significantly different, with the exception of comparisons with silent promoters from genomic background

Supplementary Figure 2

Comparison of machine learning methods

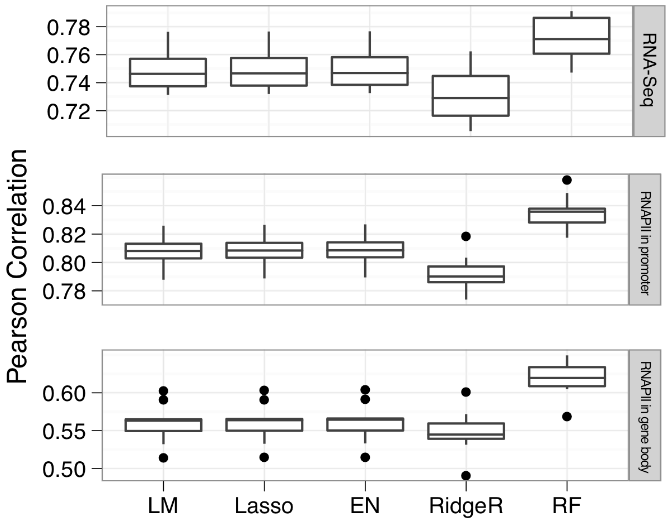

Supplementary Figure 2

The plot shows the Pearson correlations achieved between the observed and predicted promoter usage measurements. It is conditioned on different methods for measuring the usage and shows results for ordinary linear models (LM), random forest (RF) and three penalized linear models: lasso, elastic net (EN) and ridge regression. The variation estimates are achieved performing a 25% holdout experiment on 20 random splits. See **Table S1** for significance scores.

Supplementary Figure 3

Comparison of the regression results when using different gene body and promoter definitions.

A)

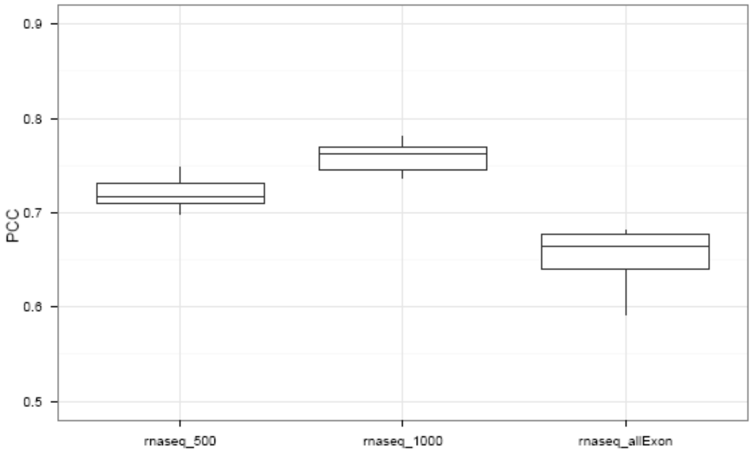

B)

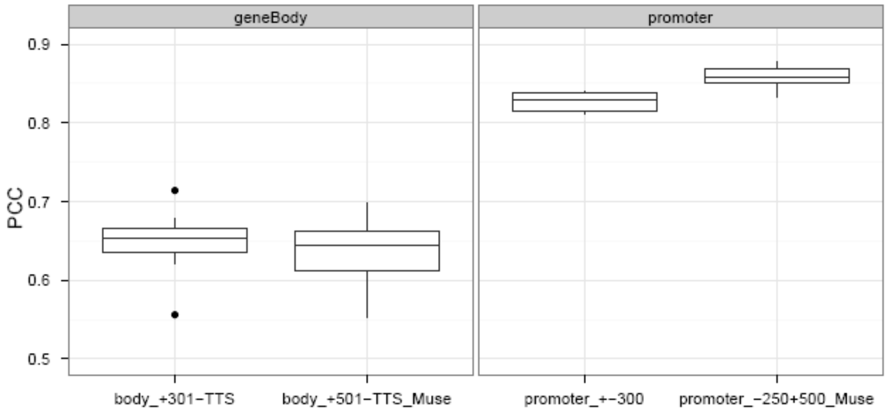

Supplementary Figure 3

A) The purpose of this analysis is to show if the use of different regions for measuring the RNA-Seq will influence the results of predicting the mRNA levels. We test three different regions for measuring RNA-Seq, either using the first 500bp exonic nt, the first 1000 nt or all the exonic nt. As mentioned above, the PCCs are computed between observed and predicted mRNA expression. Although the differences between the regions are statistical significantly different(**Table S6)**, the absolute PCCs do not vary much (~0.04-0.1 in terms of average PCCs). The difference, as discussed in Methods, might be due to the influence from alternative promoters and downstream alternative splice forms.

B) The purpose of this analysis is to show if changing the regions for measuring recruited RNAPII (core promoter region) and elongating RNAPII (gene body) will influence the results. In the left panel we compare our definition of the gene body region (left) with the one used by Muse et al [2] (right). In the right panel we compare our definition of the core promoter region (left) with the one used by Muse et al [2] (right)The results are very similar in terms of the absolute values (~0.01-0.02 difference in mean PCCs) although the difference for the recruited RNAPII is statistically significant (**Table S7**).

Supplementary Figure 4

Comparison of the linear model performance with and without the interactions between epigenetic signals

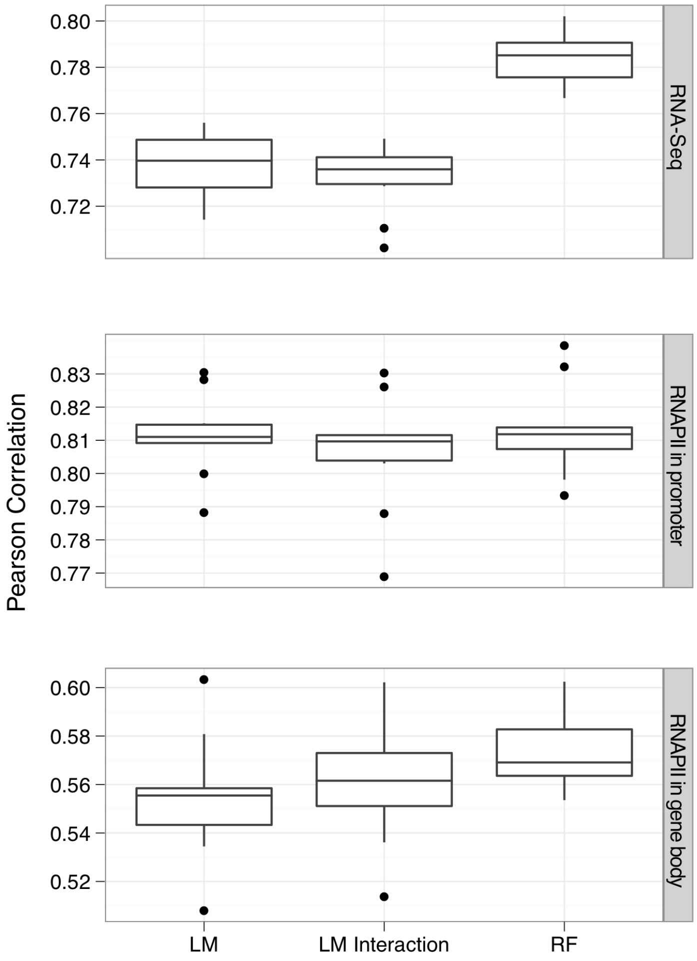

Supplementary Figure 4

The aim of this analysis was to see if the linear model framework could be improved by considering interactions between histone modifications (the Random Forest method incorporates interactions automatically). See main text for details. The PCC distribution of the linear model is at best only improved slightly by the inclusion of interactions.

Supplementary Figure 5

Assessing the necessity of H3K4me2, H3k4me3, H3k9ac and H3k27ac in RNAPII recruitment by using different thresholds and by repeating the analysis in different cell lines

A)

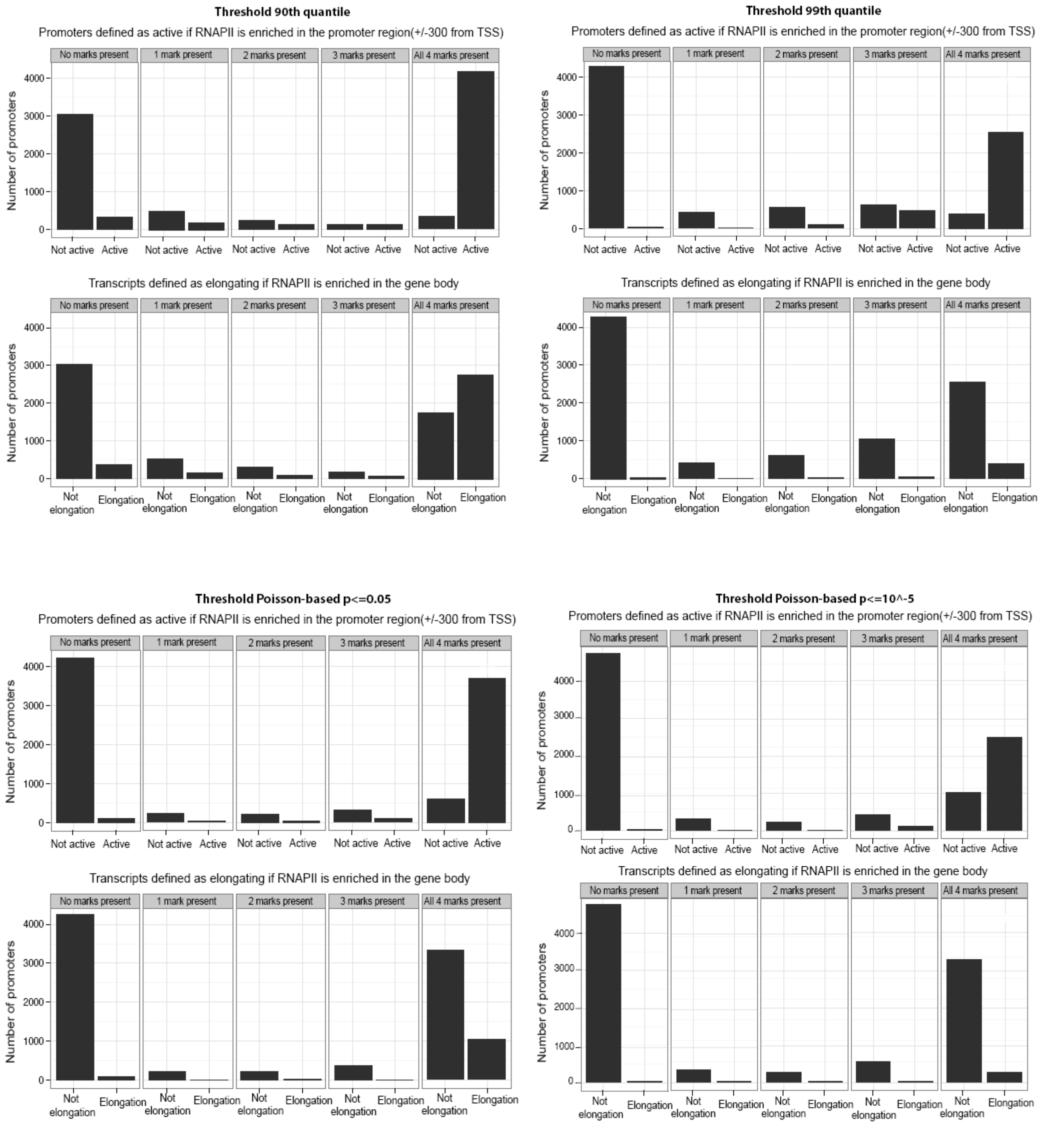

Supplementary Figure 5 (continued)

B)

Huvec

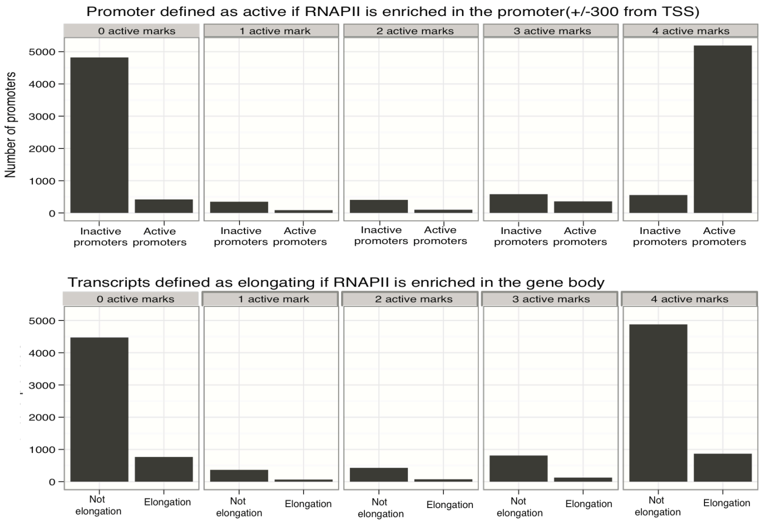

Nhek

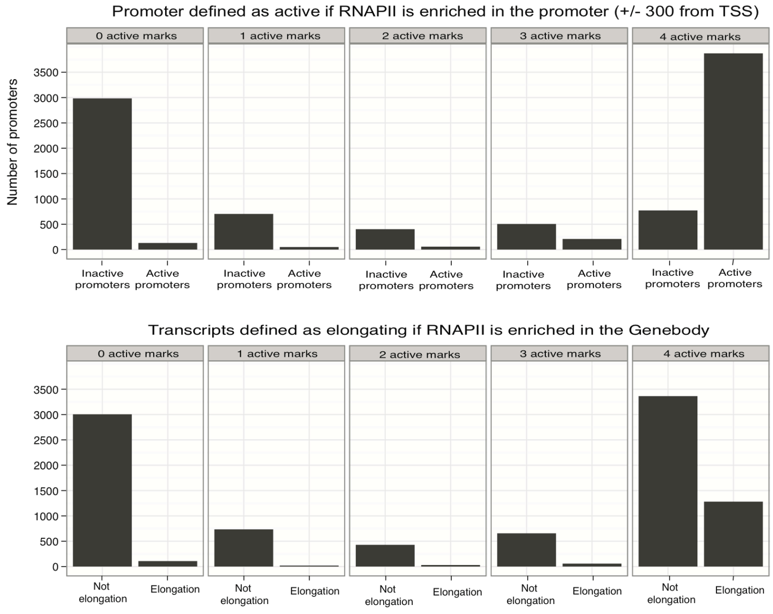

Supplementary Figure 5

A) This analysis complements **Figure 3** by using different thresholds for binarizing the presence of RNAPII in the core promoter and in the downstream gene body. The thresholds for the presence of the four histone marks also changes according to the figure header. The bottom right figure uses the same threshold as Ernst et al*[17]*. Although the actual number of RNAPII present/absent calls changes according to different thresholds used above, the trend that these four marks preferentially co-occur with the recruited RNAPII but not the downstream elongating RNAPII is reproducible.

B) The same analysis as Figure 3 but using Huvec (top figure) and Nhek (bottom figure) ENCODE data instead of K562. As above, the same pattern is observed, showing that the property is general.

Supplementary Figure 6

Correlations between histone marks

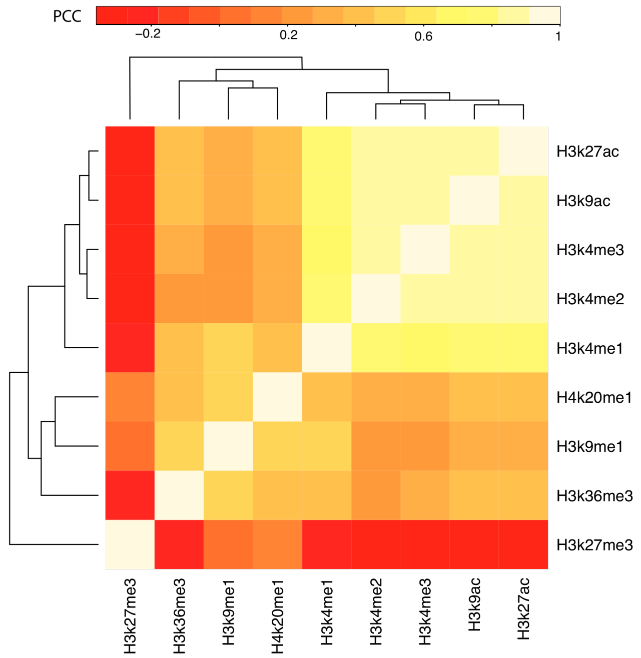

Supplementary Figure 6

The histone marks are clustered using the Spearman correlation between each pair based on the number of tags in the promoter region of respective mark. The color scale goes from red to bright yellow, where red means negative and yellow positive correlation.

Supplementary Figure 7

Distribution of stalling index given different expression level classes

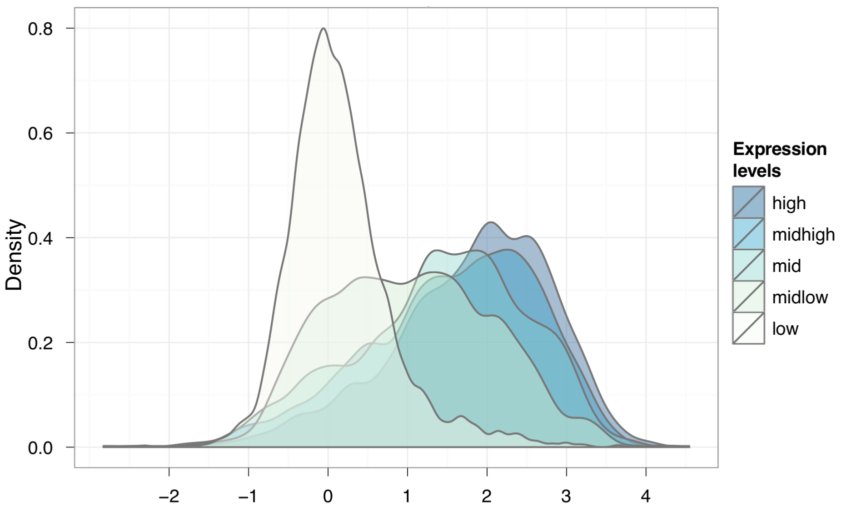

Supplementary Figure 7

We divided the promoters into classes using RNA-Seq expression and plotted the density of the S-index for different sub sets. The S-index of the silent/low expression group shows a bell-shaped distribution around zero. Promoter-proximal enrichment of polymerase (PPEP) occurs in the S-index range from 2 to 4. As mentioned by Muse et. al [2] the S index is higher in general for highly expressed genes.

Supplementary Figure 8

Importance of additional features in S-index prediction

A)

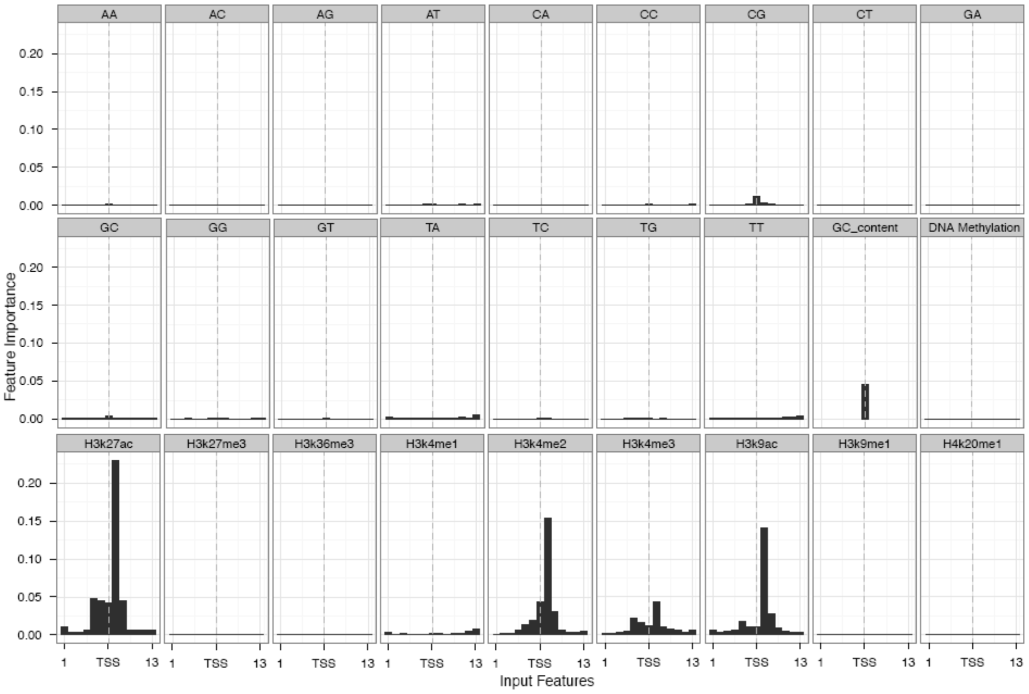

B)

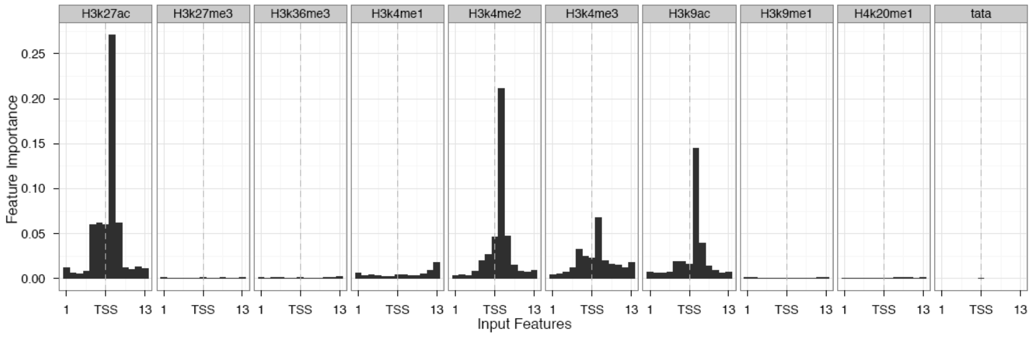

Supplementary Figure 8

A) The plot shows the importance of the input features in prediction of the S-index, using both histone modifications and additional features (dinucleotide counts, GC content and DNA methylation) (see **Figure 7B** for details). The figure represents the limited predictive power of additional features in contrast to those of histone modifications.

B) Similar to the plot above, but shows the importance estimated from the model using histone modifications and TATA box indicators (see Methods). The result demonstrates the upstream core promoter domain TATA is not as informative as the surrounding chromatin signals.

Supplementary Figure 9

Prediction of S-Index in subsets broken up by mRNA expression

A)

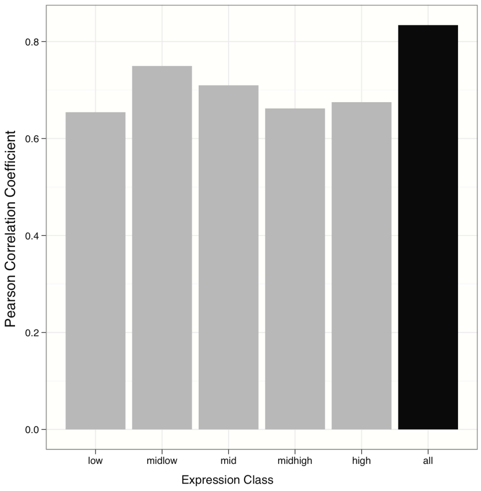

B)

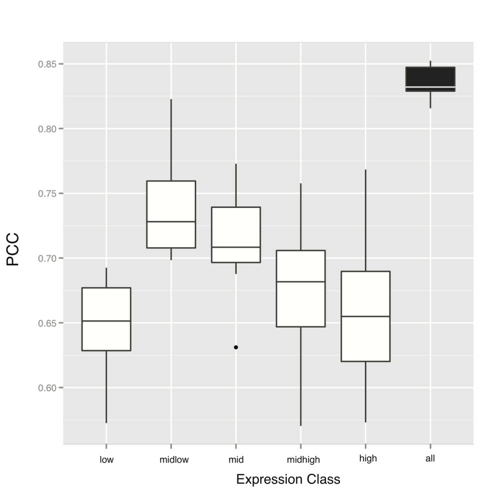

Supplementary Figure 9

A) In order to examine whether the expression levels bias our prediction results, we trained a regression model using 70% of promoters regardless of their expression, and used this to predict the S-index of the remaining promoters broken up by expression (5 groups). We assessed the accuracy by Pearson correlations, either splitting up the promoters up by the expression (grey bars) or by pooling the promoters for comparison (black bar). There is in general no substantial difference in predictive accuracies between the groups.

B) Instead of using a global model, we re-trained and re-tested our model on the different subsets independently. We obtained a median of PCC ranging from 0.65-0.73 in different sub groups based on their RNA-Seq expression levels. As above, the small fluctuation between groups indicates that our prediction on S-index do not rely on the expression levels.

Supplementary Figure 10

Prediction of S-Index in HUVEC cell line by the model trained in K562

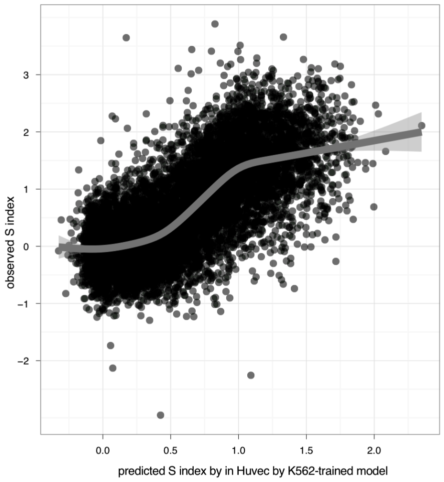

Supplementary Figure 10

We predicted the S index in HUVEC cell line using the model trained in K562 and reached a PCC of 0.71, suggesting our framework of S-index prediction is not over-fitted to the K562 data.

Supplementary Table 1

Significance of the difference between the Random Forest (**RF**) and the linear models: **ridge-**, **lasso-** and elastic net (**EN**) regression. P-values were calculated by t-tests between the PCC variance estimated in **Figure S2**.

Type of measurement	Pair of Methods	P Value
RNA-Seq	en_lasso	0.94
RNA-Seq	en_lm	0.88
RNA-Seq	en_rf	0.0028
RNA-Seq	en_ridge	0.028
RNA-Seq	lasso_lm	0.94
RNA-Seq	lasso_rf	0.0024
RNA-Seq	lasso_ridge	0.032
RNA-Seq	lm_rf	0.0020
RNA-Seq	lm_ridge	0.037
RNA-Seq	rf_ridge	6.05E-05
RNAPII in promoter	en_lasso	0.95
RNAPII in promoter	en_lm	0.88
RNAPII in promoter	en_rf	6.54E-05
RNAPII in promoter	en_ridge	0.0056
RNAPII in promoter	lasso_lm	0.93
RNAPII in promoter	lasso_rf	5.89E-05
RNAPII in promoter	lasso_ridge	0.0065
RNAPII in promoter	lm_rf	4.95E-05
RNAPII in promoter	lm_ridge	0.008
RNAPII in promoter	rf_ridge	2.17E-07
RNAPII in gene body	en_lasso	0.97
RNAPII in gene body	en_lm	0.93
RNAPII in gene body	en_rf	4.86E-05
RNAPII in gene body	en_ridge	0.29
RNAPII in gene body	lasso_lm	0.96
RNAPII in gene body	lasso_rf	4.30E-05
RNAPII in gene body	lasso_ridge	0.31
RNAPII in gene body	lm_rf	3.84E-05
RNAPII in gene body	lm_ridge	0.33
RNAPII in gene body	rf_ridge	1.06E-05

Supplementary Table 2

Significance of the difference between the regression results by using either normalized positional distribution or absolute signal strength (see Methods). P values are achieved from applying t-tests on the PCCs calculated from the cross-validation procedure.

Input Features	Type of Measurement	P Value
Signal Strength Vs Normalized Shape of the distribution	RNA-Seq	1.46E-08
	RNAPII in promoter	8.56E-06
	RNAPII in gene body	5.72E-11

Supplementary Table 3

Abbreviations:

HM: 9 Histone modifications

HM_Extra: Histone modifications and additional features (DNA methylation, dinucleotides counts, GC content)

HM_Norm: Normalized positional distribution

A) Mean AUC values for the pairwise classifiers between active promoters, silent promoters and random genomic regions.

Data Sets Compared	Feature Set	2 bins	13 bins	27 bins
Active vs silent	hm	0.969951761	0.973162844	0.970231284
Active vs silent	hm_norm	0.906103757	0.968889482	0.968375579
Active vs silent	hm_extra	0.975013409	0.975075203	0.975354
Active vs random	hm	0.975636522	0.974564853	0.972866728
Active vs random	hm_norm	0.928897052	0.967644372	0.968696573
Silent vs random	hm	0.807436787	0.795128034	0.787736726
Silent vs random	hm_norm	0.659306739	0.77613167	0.776369291

Supplementary Table 3 (continued)

B) Mean PCC values for the regression of the promoter usage based on different measurements, by using either an ordinary linear model or Random Forest.The three last columns indicate a particular measurement that is used for training and prediction.

Bins used	Feature	Model	RNAPII in Gene Body	RNAPII in Promoter	RNA-Seq
1	hm_extra	LM	0.609234438	0.810392012	0.703661609
1	hm_extra	RF	0.674031071	0.810430611	0.726557338
1	hm	LM	0.52391956	0.799244449	0.691607884
1	hm	RF	0.635483477	0.798556941	0.719134656
2	hm_extra	LM	0.595203746	0.811304333	0.727874061
2	hm_extra	RF	0.692104865	0.818758981	0.755956232
2	hm	LM	0.495317027	0.80642579	0.715740013
2	hm	RF	0.64009035	0.807343307	0.749901858
2	hm_norm	LM	0.185543064	0.371114093	0.405775141
2	hm_norm	RF	0.207120231	0.611967236	0.535704073
13	hm_extra	LM	0.603553666	0.820374083	0.729181076
13	hm_extra	RF	0.68641096	0.831443056	0.75787848
13	hm	LM	0.536118825	0.821802431	0.732392524
13	hm	RF	0.649001752	0.827016922	0.759198407
13	hm_norm	LM	0.261098145	0.554513461	0.519008042
13	hm_norm	RF	0.4085252	0.767031787	0.671767687
27	hm_extra	LM	0.59204855	0.799333892	0.711133695
27	hm_extra	RF	0.665912833	0.828498694	0.756721709
27	hm	LM	0.545556373	0.815290547	0.731479695
27	hm	RF	0.632614201	0.824803083	0.755477384
27	hm_norm	LM	0.24381834	0.50664979	0.477853136
27	hm_norm	RF	0.447822891	0.774989558	0.683867497

Supplementary Table 4

Significance of the difference between regression models using either histone modifications, or histone modifications plus additional features, including GC content, dinucleotide counts and DNA methylation (see Methods). P values are achieved from t-tests on PCCs calculated from the cross-validation.

Input Features	Type of Measuement	P value
Histone modification Vs Histone modification and additional features	RNA-Seq	0.82
	RNAPII in promoter	0.44
	RNAPII in gene body	0.05

Supplementary Table 5

Abbreviations:

HM: 9 Histone modifications

HM_TF: Histone modifications and transcriptional factors (C-myc and Nelf)

HM_Extra: Histone modifications and additional features (DNA methylation, dinucleotides counts, GC content)

HM_TATA: Histone modifications and TATA box binary indicator

TF: only input C-myc and Nelf signals

Significance of the difference between the predictions of S-index using different input features.

Feature A	Feature B	P value
hm_tf	hm	0.59
hm_extra	hm	0.0079
hm_tata	hm	0.87
tf	hm	7.16E-14
hm_extra	hm_tf	0.02
hm_tata	hm_tf	0.47
tf	hm_tf	1.03E-13
hm_tata	hm_extra	0.0037
tf	hm_extra	6.99E-13
hm	hm_tata	0.87
tf	hm_tata	1.25E-13

Supplementary Table 6

Significance of the difference between the regression models for predicting the promoter usage measured by RNA-Seq within the first 500bp exonic, 1000bp exonic or all exonic regions for each TSS. P values are achieved from conducting a t-test on the lists of PCCs computed from the cross-validation procedures.

Type of Measurement	Threshold of the Region	P Value
RNA-Seq	500bp vs 1000bp	2.60e-05
	500bp vs all exons	3.00e-05
	1000bp vs all exons	1.63e-07

Supplementary Table 7

Significance of the difference between the regression models for predicting recruited RNAPII (RNPII in the promoter) or elongating RNAPII (RNAPII in the gene body) measured in different regions -- our thresholds and the thresholds used by Muse et al [2] (see Methods for details). P values are achieved from doing t-tests on the lists of PCCs computed from the cross-validation procedures, respectively for recruited RNAPII and for elongating RNAPII.

Type of Measurement	Threshold of the Region	P value
RNAPII in the promoter	+-300 bp vs -200~+500	7.45069736993979e-05
RNAPII in the gene body	+301~TTS vs +501~TTS	0.588991868713348

Supplementary References

1. Hanley JA, McNeil BJ: **The meaning and use of the area under a receiver operating characteristic (ROC) curve**. *Radiology* 1982, **143**(1):29-36.
2. Muse GW, Gilchrist DA, Nechaev S, Shah R, Parker JS, Grissom SF, Zeitlinger J, Adelman K: **RNA polymerase is poised for activation across the genome**. *Nature genetics* 2007, **39**(12):1507-1511.
